# P-2092. Clinical Patterns Guiding Antibiotic Decision-Making in Paediatric Pyrexia of Unknown Origin (PUO) : Evidence from a Government-Funded Tertiary care hospital in a LMIC

**DOI:** 10.1093/ofid/ofaf695.2256

**Published:** 2026-01-11

**Authors:** Rithik Dharan, Sarika Karunakara Ramesh, R Sanjai, Shobana Selvaganesan, Jaflin Selcia, Shibi Selvaraj

**Affiliations:** The Tamil Nadu Dr. MGR Medical University, Chennai, Tamil Nadu, India; Jaya College of Paramedical Sciences, Chennai, Tamil Nadu, India; Jaya college of paramedical sciences, chennai, Tamil Nadu, India; Jaya college of paramedical sciences, chennai, Tamil Nadu, India; Jaya college of paramedical sciences, chennai, Tamil Nadu, India; Jaya college of paramedical sciences, chennai, Tamil Nadu, India

## Abstract

**Background:**

Paediatric Pyrexia of Unknown Origin (PUO) poses a significant diagnostic and management challenge in low-resource settings where advanced investigations are often unavailable. In such contexts, empirical antibiotic use is frequently guided by clinical judgment rather than confirmatory tests, risking both overtreatment and missed infections. This study explores symptom patterns prompting antibiotic initiation and compares outcomes in treated vs. untreated children to inform rational, resource-sensitive management.

**Figure:**
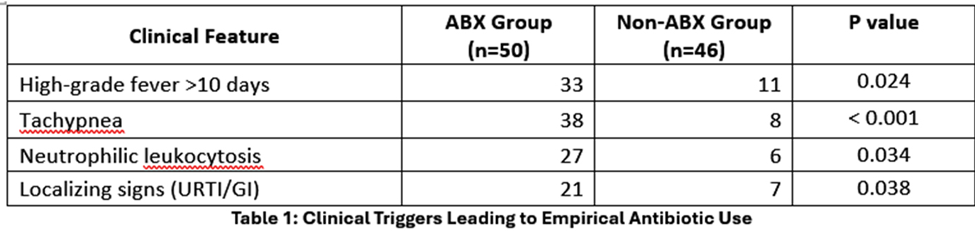

**Figure:**
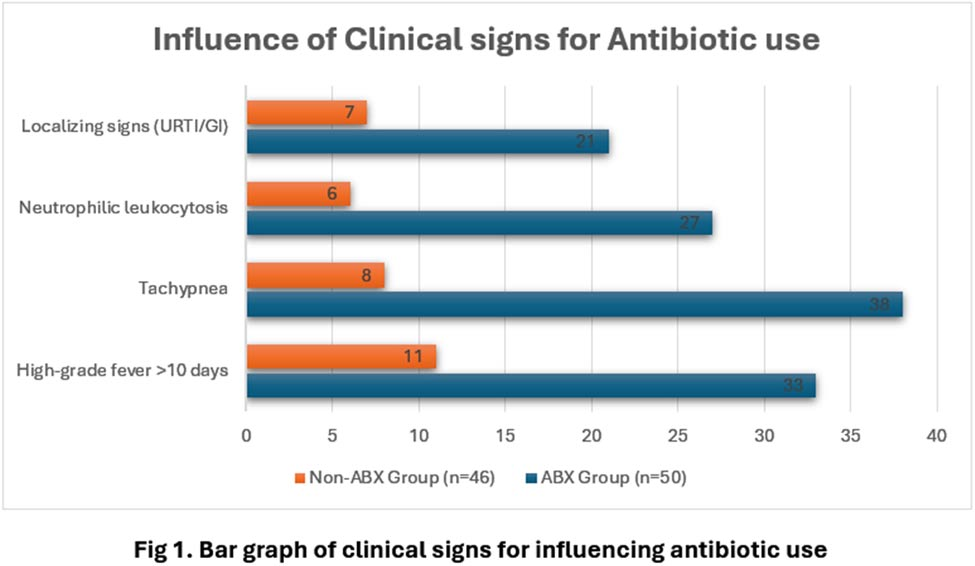

**Methods:**

This 2 month prospective observational study included 96 children 1–12 years admitted with fever > 9 days to a government-funded tertiary hospital with limited diagnostic infrastructure. Clinical features, basic labs, treatment decisions, and outcomes were recorded. Patients were grouped into those who received antibiotics (ABX) and those who did not (Non-ABX). Chi-square test and Mann-Whitney U test were used to compare categorical and continuous variables, respectively & P values < 0.05 considered significant.

**Figure:**
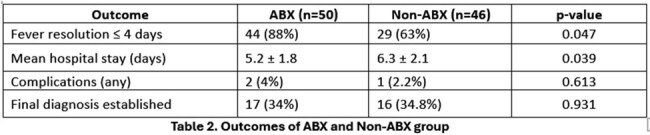

**Figure:**
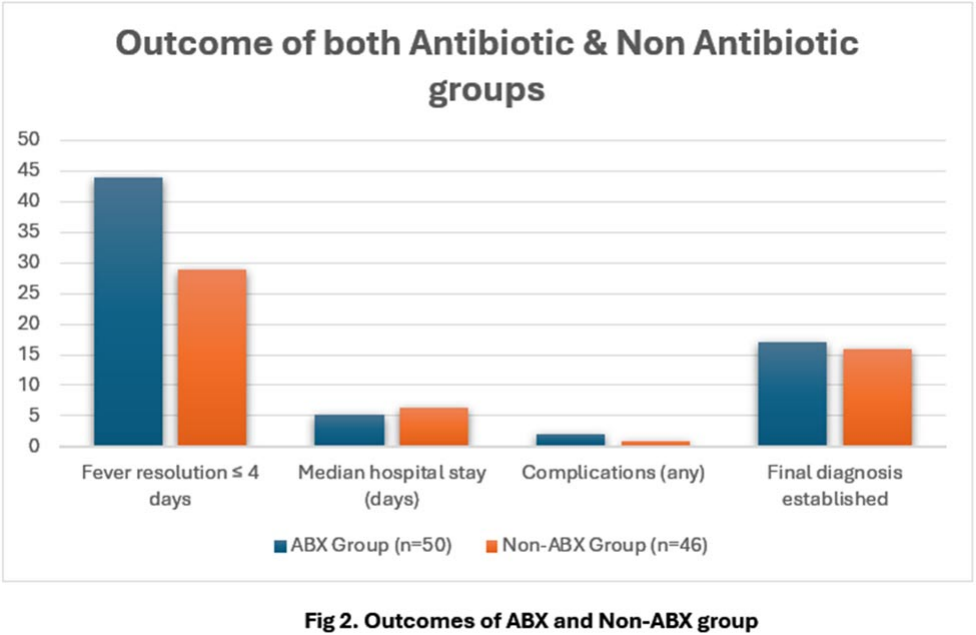

**Results:**

Among the 96 cases, 50 (52.1%) received empirical antibiotics while 46 (47.9%) did not. Clinical triggers such as high-grade fever >10 days, tachypnoea, and neutrophilic leukocytosis significantly influenced antibiotic initiation (Table 1). In terms of outcomes (Table 2), the ABX group showed faster fever resolution and shorter hospital stay compared to the Non-ABX group. No significant difference was found in complication rates or diagnostic yield.

**Conclusion:**

This study offers practical guidance for managing paediatric PUO in low-resource, government-funded hospitals. Clinical features such as tachypnoea, prolonged fever, and neutrophilic leucocytosis were key factors influencing the decision to start antibiotics, and initiating treatment based on these features was significantly associated with earlier fever resolution and shorter hospital stay. These findings highlight the value of symptom-based decision-making in settings with limited diagnostics and support the need for simple, standardized protocols to guide antibiotic use in PUO.

**Disclosures:**

All Authors: No reported disclosures

